# The Prevalence of the Frailty Syndrome in a Hospital Setting—Is Its Diagnosis a Challenge? A Comparison of Four Frailty Scales in a Cross-Sectional Study

**DOI:** 10.3390/jcm13010086

**Published:** 2023-12-23

**Authors:** Agnieszka Kasiukiewicz, Zyta Beata Wojszel

**Affiliations:** 1 Department of Geriatrics, Medical University of Bialystok, 15-089 Bialystok, Poland; zyta.wojszel@umb.edu.pl; 2 Department of Geriatrics, Marian Zyndram Koscialkowski Hospital of the Ministry of Interior and Administration in Bialystok, 15-471 Bialystok, Poland

**Keywords:** older patients, hospital acute care units, sub-intensive care units, clinical challenges, medical complexity, frailty syndrome, frailty diagnosis

## Abstract

The study aimed to assess the prevalence of the frailty syndrome in older patients hospitalized in the geriatric ward depending on the diagnostic criteria used, the feasibility of particular diagnostic scales in hospitalized patients, and their compatibility; 416 patients (81.2 ± 6.91 years) admitted to the Department of Geriatrics of Hospital of the Ministry of the Interior and Administration in Bialystok within eight months were included in the study. Four diagnostic scales were used to identify the frailty syndrome: Fried criteria, 7-point Clinical Frailty Scale (CFS), 40-item Frailty Index (FI), and FRAIL Scale. Depending on the scale, the prevalence of frailty syndrome varied from 26.8% (FRAIL Scale), 52.3% (Clinical Frailty Scale), and 58.1% (Fried criteria) up to 62.9% (Frailty Index). We observed the highest feasibility for CFS (100%) and the lowest for the Fried scale (79.8%). The highest level of agreement was found between the CFS and Frailty Index, with 80.3% consistent ratings (Cohen Kappa 0.6). Patients in the geriatric ward are characterized by a high prevalence of frailty, although it differs depending on the criteria. The most difficult to use in daily practice was the Fried scale, while the Clinical Frailty Scale was determined feasible in all patients.

## 1. Introduction

Frailty syndrome is one of the markers of unsuccessful aging. This term describes the part of older patients most susceptible to all body changes and the external environment’s adverse impact, and thus the most vulnerable to disease complications and side effects of medical therapy. This syndrome is additionally one of the main risk factors for physical and mental disability, hospitalization, and increased mortality [1].

The generally accepted pathophysiological concept of frailty syndrome is “the exhaustion of the body’s physiological reserves and the inability to maintain homeostasis in response to stressful environmental stimuli” [2]. There are two main concepts of this phenomenon. The first one treats frailty as a unique pathological process (Fried’s model) in which sarcopenia is considered to be the decisive factor underlying pathophysiological changes and an increase in sensitivity to environmental stressors. Sarcopenia additionally leads to the development or worsening of malnutrition, weight loss, and energy and protein deficiencies. It intensifies disorders of cellular processes, endocrine, and immune systems. The deficit accumulation model (Rockwood’s model) is based on the assumption that the development of frailty is influenced not by a specific disorder but by the overlapping of unfavorable factors, diseases, and psychological and social issues at the same time, which leads to disruption of the body’s homeostasis. The more unfavorable factors acting on one organism simultaneously, the more difficult it is to maintain this balance.

Frailty syndrome is a recognized risk factor for adverse health consequences, including disability and death. In recent years, many publications have described its impact on patients with cardiovascular disorders, in particular, heart failure [3,4,5], with common pathways leading to malnutrition and sarcopenia. It has also been shown that patients with frailty are less likely to receive complete pharmacological treatment recommended by medical standards (ACE inhibitors, beta-blockers), are less often qualified for coronary angiography or cardiac surgery, and have worse blood pressure control during pharmacological treatment. Much attention is also paid to oncologic patients and coexisting frailty syndrome. The last meta-analysis of 19,522 patients with breast cancer showed that frailty was associated with worse outcomes, especially in non-metastatic and younger women [6]. Therefore, more and more attention is paid to its diagnosis. European guidelines for management in many areas consider its occurrence in their algorithms and emphasize the influence of frailty on treatment and prognosis. However, this syndrome has no standardized and commonly accepted diagnostic scheme. A study assessing the issue of frailty diagnostics in everyday clinical practice confirmed that most of the doctors interviewed (83.6% of geriatricians from European countries) included frailty in the overall geriatric assessment but used different diagnostic methods [7]. The variety of scales means the same patient can be classified differently, depending on the technique [8].

Most of the published epidemiological studies on the prevalence of frailty syndrome were conducted among older adults living in an environment who are usually less comorbid and disabled than a typical geriatric patient. Hence, it is possible to underestimate the scale of the problem by following these percentages and extrapolating them to older people requiring contact with medical care. However, more surveys have been conducted on the hospitalized population in recent years—a systematic review and meta-analysis of 467,779 geriatric hospital inpatients showed the pooled prevalence of frailty as 47.4% but emphasized high heterogeneity based on clinical and demographic measurements [9]. The variety of available scales and diagnostic criteria hinders the epidemiological assessment of this phenomenon. It is not fully known which can and should be used in everyday clinical practice among older patients. Some diagnostic scales are time-consuming or require special patient assessment and may not be possible to be assessed in daily routine, especially in acute settings.

The study aimed to evaluate

1. Prevalence of the frailty syndrome in older patients hospitalized in the geriatric ward depending on the diagnostic criteria used,
2. Feasibility of diagnostic scales of frailty syndrome in hospitalized patients, sensitivity and specificity of particular criteria in diagnosing frailty syndrome.
3. Compatibility between the diagnostic scales used for frailty syndrome.
4. Association of frailty, disability, and multimorbidity in patients hospitalized in a geriatric ward.

## 2. Materials and Methods

### 2.1. Patient and Setting Characteristics

All patients admitted consecutively to the Department of Geriatrics, Hospital of the Ministry of Interior and Administration in Bialystok, Poland, in 8 months were included in the study. The Department of Geriatrics is a subacute ward, admitting patients over 60 years old, mainly with multimorbidity and functional decline; a small percentage are patients admitted from the Emergency Department with a significant deterioration of health and psycho-physical condition. A geriatric team (consisting of geriatricians, nurses, physiotherapists, and psychologists) provides a comprehensive geriatric assessment and creates a care plan.

### 2.2. Measurements

Based on the comprehensive geriatric assessment, the following data were collected:

– sociodemographic—age, sex, education, place of residence (urban/rural), the fact of institutionalization,
– clinical—weight (kg), height (cm), BMI (kg/m^2^), midarm and calf circumference (cm)—continuous variables; the presence of chronic diseases (yes/no: peripheral arterial disease, ischemic heart disease, chronic heart failure, hypertension, atrial fibrillation, history of transient ischemic attack (TIA) or stroke, chronic obstructive pulmonary disease, diabetes, neoplasm, dementia, parkinsonism, chronic osteoarthritis, osteoporosis, and chronic renal disease), medicines taken before hospitalization, nutritional condition (Mini Nutritional Assessment—Short Form [10] 8–11 points out of 14 indicate the risk of malnutrition, and 7 or less—malnutrition)
– functional—the ability to perform basic activities of daily living assessed with the Barthel index (0–100 points; a decreasing score indicates an increasing degree of patient’s disability [11]), instrumental activities of daily living (IADL) set with six items of the Duke Older American Resources and Services (OARS) I-ADL (0–12 points; a reduced number of points indicates the severity of the patient’s disability [12]), the risk of falls assessed with Timed Up and Go Test (the time taken to get up from a chair, walk a distance of 3 m at a normal pace and turn 180 degrees, return and sit on the chair is assessed; a task completion time of more than 14 s indicates an increased risk of falls [13]) and Tinetti Performance-Oriented Mobility Assessment (POMA—0–28 points; the performance of 16 tasks is assessed (9 assessing the ability to maintain balance and 7 assessing gait), a score below 26 points indicates a risk of falls) [14].
– mental—the screening scale AMTS by Hodgkinson (Abbreviated Mental Test Score) was used to assess cognitive functions (a 10-point scale consisting of 10 tasks, in which 1 point is awarded for each correct answer; a score of 7–10 points is considered normal, a score of 4–6 points indicates moderate dementia and a score of 3 or less indicates severe dementia. [15]), and in the case of reduced scores, the Folstein Mental State Examination Short MMSE (Mini-Mental State Examination) was performed (a 30-point scale; a normal result is considered as 27–30 points, in case of mild cognitive impairment the patient scores 24–26 points; 23 or less points may indicate the presence of dementia, and its severity can be additionally assessed based on the number of points: mild dementia—19–23 points, moderate dementia—11–18 points, advanced dementia—<10 points [16]). The diagnosis of dementia was made based on the overall assessment of the patient combined with an interview with an independent informant, following the recommendations of the team of experts of the Polish Alzheimer’s Society. The assessment of a clinical psychologist was also used. The risk of the depressive syndrome was assessed based on the 15-point GDS Geriatric Depression Scale (a good emotional state is indicated by a score of ≤5 points, a suspicion of moderate depression—by a score of 6–10 points, and a suspicion of severe depression—by a score of ≥11 points [17]).

### 2.3. Frailty Syndrome Assessment

Four scales were selected for the analysis, reflecting the main approaches to diagnosing frailty: the phenotypic model (Fried criteria), a model based on the examiner’s judgment (Clinical Frailty Scale), a multivariate model including—in addition to physical “fragility”—other levels, such as psychological, social, medical (FRAIL scale) and the model of accumulation of deficits (Frailty Index). The criteria were modified so that they could be applied in daily practice during the comprehensive geriatric assessment (CGA) of all patients admitted to the ward, which would also show whether they are evaluable during the routine work of the ward.

#### 2.3.1. Fried Criteria

We used diagnostic criteria for frailty syndrome according to Fried [18], based on the presence of the following symptoms:

1. unintentional weight loss (>5 kg in 12 months)—data obtained based on the patient’s or caregiver’s interview;
2. weakness—assessed based on hand grip strength measured with the SAEHAN DHD-1 hand dynamometer, taking into account sex and BMI value; the hand grip strength was measured twice for each of the upper limbs, and the highest value obtained was taken into account;
3. exhaustion—determined based on a negative answer to the question: “Do you feel full of energy?” on the Geriatric Depression Scale;
4. gait slowdown—measured by the speed of walking a distance of 15 feet (4.6 m), taking into account the sex and height of the tested person;
5. reduced physical activity—based on the 6-point Grimby scale [19], a grade of 4 and higher indicates low physical activity.

A patient with 3–5 symptoms above was classified as frail, 1–2 points—prefrail, and robust if no signs were found.

We modified Fried’s criteria for practical use in the Ward—we included the Geriatric Depression Scale (used routinely in the Department) and its question about the feeling of exhaustion, and Grimby’s Scale for assessing activity level. The Minnesota Leisure Time Activity Questionnaire, used in the original criteria, was challenging to apply to hospitalized patients, mainly since the assessed activities and physical activities differ from those performed by most older people in our community. A simple scale was therefore used, which did not require much extra time for the overall geriatric evaluation.

#### 2.3.2. Clinical Frailty Scale

The frailty syndrome was also determined in patients based on the 7-point Canadian Study of Health and Aging Clinical Frailty Scale [20], which classifies patients from category 1, i.e., high condition, to category 7—evidence of severe fragility.

To facilitate comparison between scales, patients in categories 1–3 were classified as robust, in category 4 as prefrail, and in categories 5–7 as frail, according to the literature [21].

#### 2.3.3. Rockwood’s Frailty Index

To build a scale based on the model of deficit accumulation [22], 40 variables characterizing deficits in the field of fitness and health were used: dependence on the help of other people when bathing, dressing, moving from bed to chair, moving on flat surfaces, climbing-up stairs, when eating, maintaining personal hygiene, using the toilet, dysfunction of the sphincters, difficulties with shopping, doing housework, preparing meals, disposing of money, using the phone and taking medications on your own, weight loss >5 kg in the last year, self-assessment of health, hospitalization within the last 12 months, visual and hearing impairment, difficulty in chewing food, feeling lonely, risk of depression, the presence of chronic diseases (hypertension, circulatory failure, a history of myocardial infarction, stroke, parkinsonism, cancer, diabetes, osteoarthritis, COPD, dementia), the occurrence of falls in the last year, the risk of falls measured by the Tinetti test, BMI, hand grip strength, walking speed at a distance of 4.6 m, taking more than five medications. The Frailty Index was calculated by dividing the number of factors found in a given patient by the number of measured deficits, obtaining numerical values in the range (of 0–1). Patients who lacked information in eight domains (20%) were excluded from the scale assessment [23].

To enable the comparison of the scales with each other, the respondents were also divided into 3 groups based on the Frailty Index values: FI ≤ 0.2—robust; FI—0.2–0.3—prefrail, FI ≥ 0.3—frail, under the criteria used by other authors [24].

#### 2.3.4. FRAIL Scale

The study used the diagnostic criteria of frailty syndrome based on the following symptoms [25]:

1. Exhaustion—determined based on a negative answer to the question: “Do you feel full of energy?” on the Geriatric Depression Scale;
2. Problems with climbing stairs—based on a question in the Barthel scale;
3. Problems with walking on a flat surface—based on the question in the Barthel scale;
4. Co-occurrence of more than five diseases from the following: ischemic heart disease, arterial hypertension, post-stroke condition, dementia, depression, osteoarthritis, rheumatoid arthritis, asthma, chronic obstructive pulmonary disease, diabetes, osteoporosis,
5. unintentional weight loss (>5 kg in 12 months)—data obtained from the patient’s or caregiver’s interview.

A patient with three of the above symptoms was classified as frail, with one or two symptoms—as prefrail, and if no signs were found—as robust.

### 2.4. Statistical Analysis

The STATISTICA Version 13.3 software package (TIBCO Software, Palo Alto, CA, USA) was used for statistical analyses. Variables were presented as frequency and percentage—categorical variables and medians (Me) and interquartile range (IQR)—continuous variables. The distribution of variables was checked with Shapiro–Wilk tests. Proportions were compared using χ2 tests, while the Mann–Whitney U test was used to compare medians. The compatibility assessment of the frailty syndrome diagnostic scales was performed using Cohen’s kappa statistics. The agreement was considered excellent if the kappa coefficient (weighted) was 0.81–1; good—if it was 0.61–0.8, moderate—0.41–0.6, medium—0.21–0.4, and weak—below 0.2 [26].

Feasibility was defined as the percentage of patients in the study group who could undergo testing.

Prevalence of criterion was defined as the percentage of patients with positive criterion of frailty diagnostic scale in the study group

Sensitivity was defined as the percentage of cases with positive criterion in the group of patients with frailty syndrome

Specificity was defined as the percentage of cases with negative criterion in the group of patients without frailty syndrome

These definitions from a practical and mathematical point of view are consistent with the gold standards of counting these characteristics:

Sensitivity = (truly positive/(truly positive + false negative) = truly positive/all cases with disease

Specificity = (truly negative)/(truly negative + false positive) = truly negative/all cases without disease

A two-tailed *p*-value of less than 0.05 was considered significant in all analyses.

### 2.5. Ethics Approval

The Ethics Committee approved the study at the Medical University of Bialystok (R-I-002/305/2013) on 27 June 2013. All procedures performed in the study were under the ethical standards of the Medical University of Bialystok research committee and with the Helsinki Declaration. All study participants gave their informed consent to participate in it.

## 3. Results

### 3.1. Study Cohort Characteristics

During the study period, 416 patients were hospitalized in the Geriatrics Department (Table 1). The study group consisted of 322 women and 94 men (response rate 97.4%). The mean age was 81.2 years (±6.91), median 82 (77; 86) years, minimum 60 years, and maximum 101 years; 84.1% of patients were over 75 years of age. The majority of patients lived in the city, in the environment. Widows/unmarried persons prevailed among women, while among men—married persons.

The study population was characterized by a high degree of functional disability—70% of patients reported a problem performing at least one of the basic activities of daily living, and over 80%—in one of the instrumental activities.

The gait, balance, and risk of falls were assessed using the Tinetti test (POMA) in 322 patients. For some patients, it was impossible to carry out due to the lack of cooperation or a medical condition. A risk of falls was recorded in 61.8% of respondents. The timed “Get up and Go” test, performed on 301 subjects, indicated a risk of falls in almost 65% of patients. In addition, falls over the past year were reported by over 40% of patients, similarly frequent in sex and age groups.

Patients had an average of five chronic diseases out of 15 included in the analysis, statistically significantly more men and people over 75. The most common disorders in the study population were arthrosis, diagnosed in approximately 80% of the study group, and cardiovascular diseases: arterial hypertension, ischemic heart disease, and chronic cardiac failure. In addition, more than half of the patients had decreased glomerular filtration rates, and one-third had diabetes and dementia. Patients took seven drugs on average before hospital admission—median 7 (5; 9).

### 3.2. The Frequency of the Frailty Syndrome Depending on the Diagnostic Criteria and Feasibility of the Scales

#### 3.2.1. Frailty Syndrome Diagnosed with Fried Criteria

Evaluation of frailty syndrome with Fried criteria was conducted in 389 patients. We excluded apriori patients with severe stages of dementia and MMSE < 11 points (27 persons) because of the risk of misunderstanding the questions and inappropriate performance of diagnostic tests.

As seen in Table 2, it was impossible to evaluate all the criteria of the frailty syndrome included in the scale in every patient. The most information was obtained on physical activity (the Grimby scale) and the occurrence of weight loss, and the least information was obtained on measuring walking speed. Low physical activity was the most frequently met criterion, occurring in almost ¾ of the diagnosed persons. Over 60% of the assessed patients also showed low handgrip strength and reported exhaustion. The least frequently met criterion, present in only 18.4% of respondents, was the criterion of weight loss in the last year.

All five criteria could be determined in 299 cases (76.9% of the whole group). In 33 cases, meeting three out of three specific criteria or three to four out of four criteria allowed the patient to be qualified for the frail group, and meeting one out of four specific criteria—for the prefrail group. Twenty patients (5.1%) had two or fewer criteria to investigate.

Only 28 patients were enrolled in the robust group and 111 in the prefrail group. More than half of the study group were patients with diagnosed frailty syndrome—193 patients in total, of which 15 (7.8%) met all five criteria, 94 (48.7%)—4, and 84 (43.5%)—3 diagnostic criteria of the syndrome.

Low physical activity was characterized by the highest sensitivity in diagnosing frailty, as much as 96.9%. Over 80% sensitivity also occurred in low handgrip strength, slow gait, and exhaustion. The criterion of weight loss had the lowest sensitivity of 28.1%. At the same time, this feature had the highest specificity (90.6%).

#### 3.2.2. Clinical Frailty Scale

The degree of frailty/robustness based on the CSHA Clinical Frailty Scale was possible to assess in all cases.

Patients most often presented “mild frailty”—the mode was 5 (*n* = 128), and the median was 5 (4; 5). None of the patients was classified in the first group on the CFS scale, corresponding to the best condition, i.e., active, healthy, exercising, and healthiest people in their age group. The frequency of occurrence of particular scale categories did not differ between the sex groups. However, statistically significant differences were observed in age groups (<75 years, median 5 (4; 6) vs. >75 years, median 4 (3; 5); *p* < 0.001).

After assigning individual categories to the robust group (categories 1–3), prefrail (category 4), and frail (categories 5–7), the frailty syndrome was diagnosed in 230 patients (52.3% of the study group), prefrailty—in 124 patients (29.8%), and 62 (14.9%) were qualified as robust.

#### 3.2.3. Frailty Syndrome by Frailty Index

The deficits included in the Frailty Index could be determined in most cases (Appendix A Table A1). Data gaps exceeding 5% concerned mainly information obtained from the patient’s interview (self-assessment of health status, problems with eyesight, hearing and chewing food, feeling lonely, the occurrence of falls), as well as the assessment of the risk of depression based on the GDS scale and data obtained during measurements of the handgrip and gait speed.

Determining all 40 domains of the Frailty Index was possible in 165 patients (39.7%). The analysis excludes 20 cases with missing data for more than 20% of the domains. The Frailty Index was determined in 396 patients (95.2% of the study population). The median was 0.375 (0.244; 0.513), the minimum was 0.032, and the maximum was 0.845.

The Frailty Index values were divided into three categories, robust, prefrail, and frail, under the previously defined threshold values; 17.9% of patients were classified as robust. A similar proportion was in the prefrail group, and 62.9% of the respondents were classified as frail.

The analysis of the sensitivity and specificity of particular domains in predicting frailty syndrome showed the highest sensitivity and specificity of the difficulties in instrumental activities of daily living: doing housework, shopping, and cooking. Domains related to basic activities of daily living and selected health problems (Parkinson’s disease, cancer, post-myocardial infarction, stroke, COPD) also showed high specificity but low sensitivity (Appendix A, Table A1)

#### 3.2.4. Frailty Syndrome by FRAIL Scale

Determination of the FRAIL Scale’s individual components was possible in most patients—Table 2. Data on the occurring chronic diseases were available in the entire study population. The assessment of the ability to walk on a flat surface and climb stairs was made in over 98%, based on the information contained in the Barthel scale. Information on weight loss over the past 12 months and on “exhaustion” (measured, similar to Fried, by the negative response to the Geriatric Depression Scale question for energy sense) was also collected in most cases based on data from patients’ medical records.

All five features were defined in 359 (86.3%) patients, four features—in 46 (11.1%), three features—in 9 (2.2%), and two components—in 2 (0.5%). The most frequently met criterion was exhaustion, reported by more than half of the respondents, and in the smallest percentage, the measure of multimorbidity.

According to the FRAIL Scale criteria, the frailty syndrome was determined in 373 patients (89.7% of the studied population). The syndrome was diagnosed in 100 patients (26.8%), of which one was positive for all five criteria, 26 people—4, and 73—3. The stage preceding the frailty syndrome (prefrail) was diagnosed in 186 patients (49.9%), and 87 (22.8%) patients were classified as robust (22.8%).

The highest sensitivity showed the criterion of exhaustion (98.9%) and difficulty in climbing stairs (96%). The highest specificity was recorded for inability to walk on a flat surface (94.1%) and multimorbidity (93.8%).

### 3.3. Comparison of the Diagnostic Scales

Patient assessment with the use of individual scales requires different pieces of information about the patient. Therefore, the most significant data were collected when calculating the Frailty Index. The Fried Scale and the FRAIL Scale consisted of only five criteria, and the Clinical Frailty Scale was based on the diagnosis and the researcher’s opinion.

The degree of feasibility differed for individual diagnostic scales. The assessment with the Clinical Frailty Scale could be performed in 100% of the studied population, the Frailty Index in 95.2%, and in the case of the FRAIL Scale—in 89.7%. The most difficult to apply in daily practice was the Fried Scale—it was possible only in 79.8% of the studied population.

**Figure 1:**
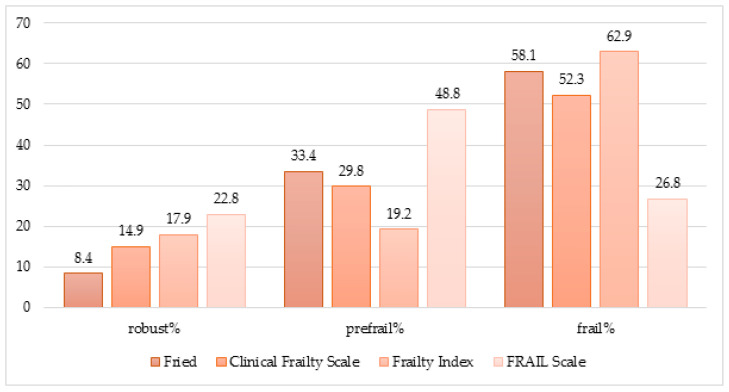
Prevalence of frailty categories depending on the diagnostic scale used. Numbers are given in percentages.

Depending on the diagnostic scale used, the frequency of the frailty syndrome in the studied group of patients ranged from 26.8% (using the FRAIL Scale) to 62.9% (based on the Frailty Index)—Figure 1. Using the FRAIL Scale, patients were classified into the frail category less frequently than in other scales, and at the same time, the percentage of the robust category was the highest in this case. On the other hand, the fewest patients were assigned to the robust category based on the Fried Scale. In 326 cases (78.4% of the studied population), it was possible to determine the presence of frailty syndrome in all four scales. In 100 cases (30.7%), these assessments were entirely consistent. Nineteen patients (5.8% of the entire study population) were assigned to the robust category, 9 (2.8%) to the prefrail category, and 72 (22.1%) patients to the frail category. Two hundred eighty-one patients were classified as frail by at least one scale.

Based on Cohen’s kappa statistic, the agreement between individual scales was assessed (Table 3). The Clinical Frailty Scale and the Fried Scale showed the highest agreement in classifying into three categories (robust, prefrail, frail), giving 70.2% concordant scores (kappa 0.49). The FI and Fried scales results were consistent in 68.9% of assessments and FI and CFS in 68.7%. The lowest agreement was found between the FRAIL Scale and the Frailty Index, with only 44.8% of concordant scores (0.2 kappa).

Higher kappa coefficients were obtained by assessing compliance in qualification using scales up to 2 categories: frail and non-frail (aggregated cases from the robust and prefrail groups). In this case, the kappa coefficient ranged from 0.6 for the Clinical Frailty Scale and Frailty Index scales to 0.31 for the FRAIL Scale and Frailty Index.

### 3.4. Frailty, Disability, and Multimorbidity

**Figure 2:**
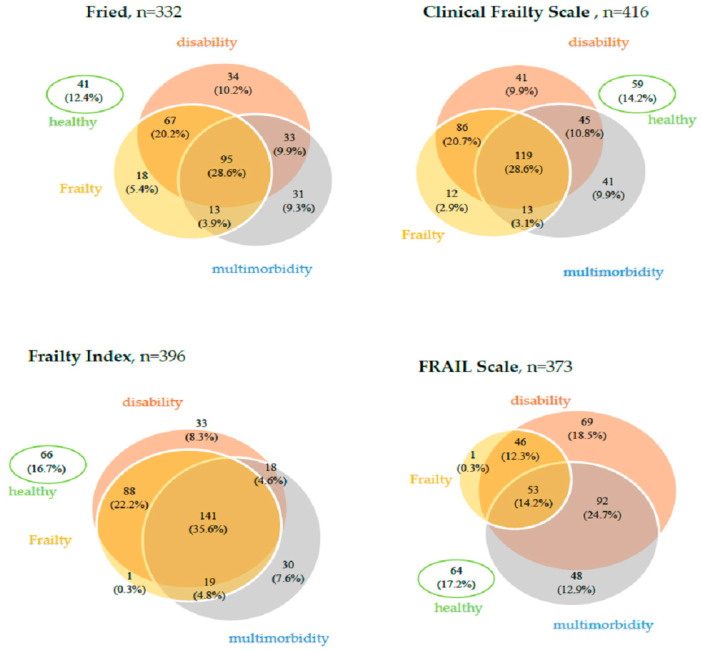
Overlapping of frailty, disability, and multimorbidity depending on the frailty diagnostic scale used.

The frailty syndrome was also associated to varying degrees with the co-occurrence of disability (defined as impairment in performing at least one activity in the scope of basic activities of daily living) and multi-morbidity (Figure 2).

Using the Fried criteria, frailty syndrome coexisted with disability in 162 cases (83.9%) and with multimorbidity in 108 cases (55.9%); 95 patients (28.6% of the group) were diagnosed with all three syndromes, 18 people (5.4%) were diagnosed with only frailty syndrome, 34 people (10.2%)—only disability, and 31 (9.3%)—only multi-morbidity.

Frailty syndrome diagnosed based on the Clinical Frailty Scale was associated with disability in 205 cases (89.1%) and with multimorbidity in 132 cases (57.4%). All three syndromes occurred in 119 patients (28.6% of the group), with only multimorbidity—in 41 (9.9%), only disability—similarly in 41 (9.9%), and only frailty syndrome—in 12 (2.9%) patients.

Using the Frailty Index, 229 (92%) of frailty cases were associated with disability, and 160 (64.3%) were associated with multimorbidity. Only one person had the frailty syndrome, and as many as 141 patients (35.6%) had all three syndromes.

Using the FRAIL Scale, frailty coexisted with disability in 99% of cases and with multimorbidity in 53% of cases. Only one patient classified as frail did not have the remaining two syndromes. Multi-morbidity, disability, and frailty co-occurred in 53 patients (14.2% of the study group).

None of the above syndromes was diagnosed in 12.4% (using the Fried criteria)—17.2% (using the FRAIL scale) of patients.

## 4. Discussion

Frailty syndrome is highly prevalent in hospitalized older adults and may affect clinical outcomes and the need for modification of medical treatment. Therefore, it is crucial to screen patients for this syndrome. We tried to address the question of whether broadly recommended scales are feasible for geriatric populations in hospital circumstances in the “real world” study. We assessed frailty in patients admitted to the geriatrics ward, highly burdened with disability and multiple diseases, and found out that, despite the diagnostic difficulties, the feasibility of all assessed scales was high. The Fried scale was the most difficult to apply (the most missing data, in this case, were observed in measuring walking speed), while the Clinical Frailty Scale could be determined in all patients.

The assessment of the applicability of the Fried criteria in hospital practice was the aim of another Polish study [27], where the prevalence of frailty syndrome was examined in 500 patients consecutively admitted to the geriatric ward (mean age 79 ± 8.4 years, 67% women). Determining all Fried criteria was possible only in 65% of the studied population. The most missing data were related to weight loss (27.4%), followed by walking speed (18.2%—mainly due to the inability to walk). In another prospective study, also carried out on a group of hospitalized patients (511 patients admitted consecutively to the geriatric ward, mean age 83.7 ± 4.8 years, 62% women) [28], the Fried criteria could not be assessed in 291 (56.9%) patients. Those significant data gaps resulted mainly from the patient’s refusal to cooperate, a severe medical condition, profound cognitive impairment, or problems with vision and hearing. Similar to our study, measuring walking speed and handgrip strength was the most difficult to determine in the remaining group of patients. The hospital departments described in the above-mentioned studies hospitalized patients with acute diseases. Hence, we can conclude that the possibility of using the phenotypic model in hospital practice is limited.

A previous Spanish study, conducted on 185 patients with a median age of 89 years, also assessed the feasibility of different diagnostic scales (FRAIL, CFS, handgrip strength, and Spanish Frailty-VIG in hospital conditions [29]. The feasibility of the instruments was 100%, except for handgrip strength (67%). This study’s prevalence of frailty varied from 65.2% (FRAIL) to 86.7% (VIG).

Our study’s frequency of meeting particular Fried criteria differed from those in previously published articles. It might result from differences between the assessed populations. For example, in the CHS Cardiovascular Health Study (the source study of the Fried criteria), the most common measure met was low physical activity. It was present in 22% of the respondents, in 20%—slowing down of walking and weakening of the handgrip strength, and only 6% of weight loss was observed. In the SHARE Study [30], carried out in the group of people over 65 living in the environment, the criterion of exhaustion (36.7%) was most often met, followed by weakness (26.3%), slowing down of walking (22.7%), low physical activity (21.3%) and weight loss (11.1%). These were, however, populations younger than the one studied in our analysis. Compared to that, in studies conducted among 100-year-olds [31], low physical activity was recorded at 78%, weakness and slowing down—at 68%, exhaustion—at 42%, and weight loss—at 6%. Differences between individual tests may also arise from using different tests to define specific criteria. For example, in a Dutch study on more than 8000 community-dwelling people over 55 (mean age 74.2 ± 6.4 years), the researchers asked about the weakening of hand strength and the ability to cross a street before changing the lights. They did not use any measuring of the walking speed and the handgrip [32].

The Clinical Frailty Scale could be used in all patients, which is undoubtedly an advantage when choosing a diagnostic scale for hospital wards. This scale grades not only “frailty” (groups 5–7) but also “robustness” (groups 1–3) and can be used in acute settings [33]. Notably, none of the respondents in the presented study was classified as group 1, which proves they were in worse health condition.

The Frailty Index assessed the most significant number of factors and domains and was thus the most time-consuming. Since most of the data came from the CGA, we could determine the individual components in most patients. Data gaps exceeding 20% of the domains included made it impossible to calculate FI values among 20 patients. On the other hand, we could define all 40 components in 39.7% of the study population. The median FI value was 0.38. It was significantly higher in our study than in the studies already described using the Frailty Index. In CSHA, the mean FI value was 0.24 ± 0.15, in SHARE-0.14 for women and 0.11 for men, and similar to the FI value calculated in a study in acute hospital wards (FI—0.32 ± 0.14) [34]. The literature uses different FI cut-offs for subdividing patients into robust, prefrail, and frail. The threshold of recognizing the frailty syndrome is often assumed to be 0.25, and for the division into robust and prefrail, it is 0.08. In the present study, slightly higher FI diagnostic values (0.2 and 0.3) utilized more often in nursing home patients—were used, bearing in mind the high degree of disability of the ward patients.

The FRAIL Scale, a simple screening tool, could also be determined in most patients, in line with the assumptions of this test’s authors. The most missing data, in this case, concerned the Geriatric Depression Scale and the question about the feeling of exhaustion. It was associated with dementia and the patient’s inability to understand the question. There were no exclusion criteria, such as the Fried scale (we did not assess patients with profound dementia). What is noteworthy is the low percentage of meeting the multi-morbidity criterion in this population, generally burdened with a high degree of disability and several chronic diseases. When calculating the FRAIL scale, with the median of conditions in the study group amounting to 5 (3; 6), the criterion of multimorbidity in the presented study was the presence of more than five diseases among those most frequently proposed in the literature. Some tests use a bar of five or more chronic disorders, and the list of medical conditions differs between them. In the study where this scale was initially used [35], the proportions between the individual positive criteria were similar as in our analysis, and multi-morbidity also had the lowest prevalence (2.1%) compared to the other ones (20.1–27.7%).

In the presented study, the frequency of the frailty syndrome ranged from 26.8% (using the FRAIL scale) to 62.9% (using the Frailty Index). Only a tiny percentage of patients could be classified as robust (from 8.4 to 22.8%, depending on the scale used). Moreover, as many as 281 patients (i.e., 67.5%) were qualified as frail by at least one of the scales used, and 72 persons were considered frail on all four scales. This percentage is significantly higher than in the CHS study population (6.9%—using the Fried criteria) or the European SHARE (6%—about 45%, depending on the diagnostic scale for the weakness syndrome). As mentioned, these studies described younger cohorts of people living in the community.

Moreover, the CHS study excluded people with Parkinson’s disease, after stroke, depression, and moderate dementia from determining the frailty phenotype. In our study, only patients with severe dementia were excluded from the Fried scale due to the patient’s lack of understanding of the instructions. Moreover, Parkinson’s syndrome and cognitive impairment or depression may predispose to the frailty syndrome.

In the other Polish studies on hospital inpatients, the incidence of frailty syndrome assessed using the Fried criteria was 54.2% [25], using the Edmonton scale—12.3% (mean age 62.6+/−9.7y) [36], and SHARE-FI (mean age 73 (68–81)y) − 39% [37]. In another Polish study, in patients hospitalized due to heart failure (mean age 71.6 ± 10.9 years), frailty frequency using the Tilburg Frailty Indicator scale was 71.62% [38]. However, despite hospitalization in geriatric wards, these were younger and more independent patients than our study population. A recent study, FRAILTOOLS, comparing frailty tools in hospitalized patients, oscillates around 60–70% [39].

Individual scales classified the same patients differently—the highest percentage of frailty syndrome was recorded using the Frailty Index, and the lowest—was with the FRAIL Scale. A similar analysis was carried out in the SHARE study using eight diagnostic scales [25], where the frailty frequency ranged from the highest using Groningen (43.9%) and Tilburg Frailty Indicator (29.2%), by Frailty Index (21.6%), Clinical Frailty Scale (16.3%), Fried criteria (11%) to only 6.1% using the FRAIL scale. Our study obtained the highest correspondence between the Fried and CFS scales (Cohen’s kappa coefficient 0.49), and the lowest was between FRAIL and the other scales (kappa 0.2–0.29). The SHARE study obtained similar results. Further studies comparing different scales with each other showed a comparable or lower agreement between the scales. For example, in a survey of the Russian population living in the environment, comparing the Fried scales, the model of deficits accumulation and Groningen resulted in an agreement (Cohen’s kappa) between 0.14 and 0.3 [40]. However, in studies on nursing home patients, the compatibility between the Fried and FI scales was only 0.17 [41]. These results are a derivative of the variety of diagnostic tools used. Partially, they may result from different purposes for which they were created (clinical, scientific, or social), so when choosing a specific scale, one should also take into account its suitability for specific “recipients”. A meta-analysis on the accuracy of different screening tools in emergency departments [42] showed that available frailty screens could accurately diagnose frailty in older adults attending. Still, specificity was comparatively low, and additional assessment may be required to identify those requiring in-hospital management. The authors conclude further study is, therefore, needed.

Of all the Fried criteria, low physical activity was characterized by the highest sensitivity but, at the same time, the most insufficient specificity, which indicates the generally low physical activity of the study population. Handgrip strength measurements were also characterized by high sensitivity and low specificity. The gait slowing criterion had the best sensitivity-to-specificity ratio. It is also reflected in the fact that gait speed measurement is used as an independent diagnostic scale for frailty syndrome, as well as the proven predictive value of this feature for the occurrence of disability over subsequent years of observation [43]. The most significant sensitivity and specificity criteria were ambulance criteria—difficulty walking on flat ground and resistance—climbing stairs, which may indicate sarcopenia and are equivalents to weakened handgrip strength and slowed gait in the phenotypic model.

The high incidence of frailty syndrome in our population can be additionally the effect of the high coincidence of multimorbidity and disability in daily activities. Frailty, disability, and multimorbidity are not the same constructs, but they overlap to a large extent. Frailty is sometimes treated as a condition preceding disability. Still, most frail people hospitalized in a geriatric ward in our study were already dependent on basic (83.9–99%) and instrumental (93.2–98.8%) activities of daily living. The analysis of older adults living in rural areas showed the co-occurrence of three syndromes in 55% and frailty—only in 9.6% of the respondents [44]. A comparison of four different frailty diagnostic tools was also carried out in the study on nursing home residents in northern Spain [45]. Regardless of the scale used, the authors found the co-occurrence of these three syndromes in approximately 50% of nursing home residents. In contrast, the frailty syndrome alone was diagnosed in the highest percentage (6.4%) when the Fried criteria were used and when the FRAIL-NH (Nursing Homes) scale was used– no such patients were found. As the above comparisons show, according to its concept, frailty syndrome differs from disability and multimorbidity. However, their overlap is often observed in populations with a higher percentage of these geriatric syndromes.

When analyzing these results, one should also bear in mind the weaknesses of this study. It aimed to determine the frequency of the frailty syndrome among typical geriatric in-patients. The study was carried out on a group of patients consecutively admitted to the geriatrics ward over eight months. Therefore, it was not a random sample, so one should interpret the presented results cautiously. Moreover, the analyses were hampered by missing data, partly due to the inability or difficulty to cooperate with patients, their serious medical condition, and technical challenges, such as a temporary lack of staff in the ward. On the other hand, the study aimed to determine the feasibility of individual diagnostic scales of the frailty syndrome in the conditions of routine hospital work, so it can be qualified as a real-world study. The main weakness of our study was the research sample. It was a single-center study, which makes it difficult to extrapolate the results to other countries, hospitals, and types of hospital wards. Subsequent studies should be conducted as multicenter studies, which could positively impact the results’ generalizability.

## 5. Conclusions

1. A high frequency of frailty syndrome characterizes geriatric ward patients, although the numbers vary depending on the diagnostic criteria. The Frailty Index found the highest incidence and the lowest using the FRAIL scale.
2. The feasibility of selected diagnostic scales based on data collected during the comprehensive geriatric assessment in everyday clinical practice differed. The Fried scale was the most difficult to apply (the most missing data, in this case, was observed in measuring walking speed), while the Clinical Frailty Scale could be determined in all patients. Despite the aforementioned diagnostic difficulties, the feasibility of all assessed scales was high.
3. Diagnostic scales of the frailty syndrome showed satisfactory agreement—the highest for the Frailty Index and Clinical Frailty Scale and the lowest for the FRAIL with the other scales.

## Figures and Tables

**Table 1:** Study group characteristics.

	Total	Sex		*p*	Age		*p*
		Women	Men		<75 Years	≥75 Years	
No. (%) of patients	416 (100)	322 (77.4)	94 (22.6)		66 (15.9)	350 (84.1)	
Age, y, M (SD)	82 (77; 86)	82 (77; 86)	82.5 (78; 87)	0.39			
Sex, women, *n* (%)					54 (81.8)	268 (76.6)	0.34
Place of residence (urban), *n* (%)	329 (79.1)	254 (78.9)	75 (79.8)	0.84	56 (84.9)	273 (78)	0.2
Barthel Index, Me (IQR)	90 (70; 100)	90 (70; 100)	95 (70; 100)	0.07	97 (90; 100)	90 (70; 95)	<0.001
Duke OARS IADL, Me (IQR)	7 (3; 11)	7 (4; 11)	7 (2; 10)	0.17	10 (7; 12)	7 (2; 10)	<0.001
POMA, Me (IQR)	23 (17; 28)	23 (17; 28)	24 (19; 28)	0.66	27.5 (23; 28)	22.5 (17; 28)	<0.001
TUG, s, Me (IQR)	17.4 (11.9; 28)	18 (12; 28)	14.7 (11.4; 28.8)	0.19	11.6 (9.5; 17)	19 (12.7; 30)	<0.001
Orthostatic hypotension, *n* (%)	57 (16.2)	32 (11.7)	25 (32.1)	<0.001	8 (14.6)	49 (16.4)	0.72
Risk of depression— GDS > 5, *n* (%)	181 (56.9)	148 (58.5)	33 (50.8)	0.26	32 (62.8)	149 (55.8)	0.35
Risk of cognitive impairment—AMTS < 6, *n* (%)	111 (29.1)	88 (29.6)	23 (27.4)	0.68	5 (8.8)	106 (32.7)	<0.001
Number of chronic diseases, Me (IQR)	5 (3; 6)	4 (3; 6)	5 (4; 7)	0.003	3.5 (2; 6)	5 (3; 6)	<0.001
Hospitalization in the last year, *n* (%)	122 (29.5)	83 (25.9)	39 (41.9)	0.002	17 (25.8)	105 (30.3)	0.46

χ2 test or U Mann–Whitney test, as appropriate; Abbreviations: IQR, interquartile range; Me, median, POMA—Performance-Oriented Mobility Assessment, TUG—Timed Up and Go test, GDS—Geriatric Depression Scale, AMTS—Abbreviated Mental Test Scoring.

**Table 2:** Feasibility of particular diagnostic criteria (%).

Criterion	Feasibility	Prevalence	Sensitivity	Specificity
Fried scale				
Gait speed	79.2	51.9	82.6	84.9
Handgrip	86.6	65.3	85.6	61.9
Exhaustion	93.1	61.6	86.7	71.7
Weight loss	97.9	18.4	28.2	90.6
Low physical activity	98.5	73.4	96.9	61.1
FRAIL scale				
Inability to walk 100 m	99.8	29.6	81.9	94.1
Inability to climb stairs	98.8	44.2	96.0	78.4
Exhaustion	87.0	53.6	98.9	50.5
Weight loss	97.6	18.3	49.0	89.7
Multimorbidity	100	7.7	13.0	90.8

Numbers are given as percentages.

**Table 3:** Compatibility between diagnostic frailty scales (Cohen’s kappa statistic).

	Conformity of Qualifications to Three Categories (Robust—Prefrail—Frail)			Conformity of Qualifications to Two Categories (Non-Frail–Frail)		
	% of Matching Answers	Weighted Kappa	95% Cl	% of Matching Answers	Weighted Kappa	95% Cl
Fried-CFS	70.2	0.49	0.45–0.53	79.2	0.58	0.53–0.63
Fried-FI	68.9	0.45	0.41–0.49	79.6	0.58	0.52–0.64
CFS-FI	68.7	0.45	0.41–0.49	80.3	0.60	0.55–0.65
FRAIL-CFS	53.1	0.29	0.26–0.32	69.7	0.41	0.37–0.45
FRAIL-Fried	48.9	0.23	0.19–0.27	64.9	0.35	0.31–0.39
FRAIL-FI	44.8	0.20	0.17–0.23	61.7	0.31	0.27–0.35

Abbreviations: CFS—Clinical Frailty Scale, FI—Frailty Index, Fried—Fried scale, FRAIL—FRAIL scale.

## Data Availability

The data supporting the results in the current study are available from the corresponding author upon reasonable request.

## Appendix A

**Table A1:** Feasibility, prevalence, sensitivity, and specificity of Frailty Index domains.

Deficit	Feasibility, *n* (%)	Prevalence, *n* (%)	Sensitivity (%)	Specificity (%)
Dependence in eating meals	413 (99.3%)	70 (16.8%)	24.9	100
Dependence in transfer from bed to chair	412 (99%)	115 (27.6%)	42.2	99.3
Dependence in grooming	413 (99.3%)	80 (19.2%)	27.7	99.3
Dependence in using toilet	413 (99.3%)	133 (32%)	49.8	100
Dependence in taking bath	411 (98.8%)	202 (48.6%)	73.1	94.6
Dependence in walking	415 (99.8%)	123 (29.6%)	44.6	99.3
Dependence in climbing stairs	411 (98.8%)	184 (44.2%)	63.1	89.1
Dependence in dressing	413 (99.3%)	137 (32.9%)	50.2	98.7
Incontinence	411 (98.8%)	Urine or stool—151 (36.3%) Urine and stool—47 (11.3%)	28.2	98.3
Dependence in housework	405 (97.4%)	304 (73.1%)	96.4	62.6
Dependence in preparing meals	405 (97.4%)	228 (54.8%)	81.5	89.1
Dependence in shopping	405 (97.4%)	279 (67.1%)	93.6	74.8
Dependence in handling money	408 (98.1%)	178 (42.8%)	63.5	92.5
Dependence in using telephone	409 (98.3%)	136 (32.7%)	49	96.6
Dependence in taking medicines	409 (98.3%)	174 (41.9%)	61	91.2
Self-assessment of health status compared to other people of similar age	359 (86.3%)	Rather better—130 (31.3%) Rather worse—159 (38.2%) Much worse—61 (14.7%)	62.5	59.2
Vision problems	374 (89.9%)	137 (32.9%)	44.6	83
Hearing problems	373 (89.7%)	117 (28.1%)	38.2	85
Chewing problems	374 (89.9%)	112 (26.9%)	35.7	84.3
Feeling lonely	368 (88.5%)	sometimes—151 (36.4%) often—95 (22.8%)	44.6	70.1
Hospitalization in last year	386 (92.8%)	122 (29.5%)	34.1	80.9
Polypharmacotherapy	407 (97.8%)	322 (77.4%)	83.9	32.6
Falls in last year	358 (86.1%)	157 (43.9%)	43.4	67.3
Hypertension	416 (100%)	327 (78.6%)	78.7	20.4
Ischemic heart disease	416 (100%)	223 (53.6%)	57.4	51.7
History of heart infarct	416 (100%)	39 (9.38%)	12.1	94.6
Heart failure	416 (100%)	162 (38.9%)	47.8	76.2
History of stroke/TIA	416 (100%)	56 (13.5%)	18.5	95.2
Diabetes	416 (100%)	126 (30.3%)	31.3	74.8
Asthma/COPD	416 (100%)	42 (10.1%)	10.8	90.5
Active neoplasm	416 (100%)	32 (7%)	7.6	93.9
Arthritis	416 (100%)	324 (77.9%)	81.9	25.8
Osteoporosis	416 (100%)	74 (17.8%)	17.3	83.7
Dementia	416 (100%)	severe-27 (6.5%); moderate-45 (10.8%); mild-64 (15.4%): MCI-44 (10.6%)	40.6	83
Parkinson’s disease	416 (100%)	55 (13.2%)	18.5	96.6
Risk of depression (GDS scale)	318 (76.4%)	181 (56.9%)	38.4	88.9
Weight loss 5 kg/last year	406 (97.6%)	76 (18.7%)	19.7	83
Low handgrip	337 (81.0%)	220 (65.3%)	69.1	60.5
Slowness of gait (4.6 m)	308 (74.0%)	160 (51.9%)	55.8	81.6
Risk of falls (POMA scale)	322 (77.4%)	199 (61.8%)	37.4	99.3

Abbreviations: TIA—Transient Ischemic Attack, COPD—Chronic Obstructive Pulmonary Disease, POMA—Performance-Oriented Mobility Assessment, GDS—Geriatric Depression Scale.
